# 334. Impact of Overall Dexamethasone Exposure on Development of Invasive Pulmonary Aspergillosis in Hospitalized Patients with COVID-19

**DOI:** 10.1093/ofid/ofab466.535

**Published:** 2021-12-04

**Authors:** Erik Skoglund, Amy Kum, Allison Mac, Mark Nguyen

**Affiliations:** 1 Western University of Health Sciences, College of Pharmacy, Los Angeles, California; 2 Dignity Health, St. Mary Medical Center, Long Beach, California

## Abstract

**Background:**

Abbreviated courses of corticosteroids, such as dexamethasone, have demonstrated significant improvements in clinical outcomes among patients infected with COVID-19, although chronic corticosteroid use can predispose patients to opportunistic infections. The RECOVERY trial investigators showed reduced 28-day mortality among patients treated with 6 mg/day dexamethasone for up to 10 days, however in clinical practice the dosage and duration of dexamethasone therapy can vary widely based on severity of disease and provider discretion. Upon observing an anecdotal increase in the number of patients presenting with potential invasive aspergillosis during the third wave of COVID-19, we sought to evaluate the impact of overall dexamethasone exposure on the development of invasive pulmonary aspergillosis.

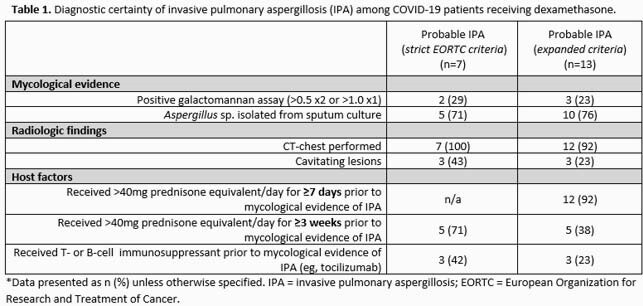

**Methods:**

Patients presenting to our institution from Dec. 2020 – Jan. 2021 with positive PCR for SARS-CoV-2 were screened for dexamethasone therapy. Assignment of high vs low dose dexamethasone groups were retrospectively made based on overall dexamethasone exposure. Low dose dexamethasone assignment was restricted to a total exposure of no more than 78 mg during a patient’s hospitalization. Adjudication of invasive pulmonary aspergillosis was made based on criteria that included host factors, radiologic findings, clinical factors, and mycological evidence.

**Results:**

Dexamethasone therapy was provided to 202 patients admitted to the hospital with COVID-19. Invasive pulmonary aspergillosis was determined to be probable in n=7 patients based on European Organization for Research and Treatment of Cancer (EORTC) criteria, and in n=13 patients based on expanded criteria. Patients in the low dose dexamethasone group were less likely to be diagnosed with probable IPA based on EORTC criteria (n=0, 0% on low dose vs. n=7, 11% on high dose) as well as expanded criteria (n=9, 5% on low dose vs. n=11, 17% on high dose), p< 0.001.

**Conclusion:**

Patients hospitalized with COVID-19 receiving high-dose dexamethasone may be at a higher risk of opportunistic infections such as invasive pulmonary aspergillosis compared to patients who receive low-dose dexamethasone therapy. Further investigation is needed to obtain higher certainty of IPA diagnosis.

**Disclosures:**

**All Authors**: No reported disclosures

